# Supplementary material for: Batch Effect Correction for Neuroimaging Data with Heterogeneous Spatial Correlations
Source: bioRxiv. 2026 Jun 8:2026.06.03.729396. Preprint. [Version 1] doi: 10.64898/2026.06.03.729396 (PMC13277852; doi:10.64898/2026.06.03.729396)
Supplement: Supplementary file 1 [file NIHPP2026.06.03.729396v1-supplement-1.pdf]

bioRxiv preprint doi: https://doi.org/10.64898/2026.06.03.729396; this version posted June 8, 2026. The copyright holder for this preprint (which was not certified by peer review) is the author/funder, who has granted bioRxiv a license to display the preprint in perpetuity. It is made available under a CC-BY 4.0 International license.

Appendix

5.1 Supplemental Calculations and Details

We first assume that Y_{ijv} can be modeled in the following way:

$$Y_{ijv} = \alpha_v + x_{ij}^T\beta_v + \gamma_{iv}^* + \delta_{iv}^* e_{ijv}$$

in which $e_{ijv} \sim N(0, \sigma_v^2)$. We first calculate the standardized data Z_{ijv} as

$$Z_{ijv} = \frac{Y_{ijv} - (\hat{\alpha}_v + x_{ij}^T\hat{\beta}_v)}{\hat{\sigma}_v}.$$

$\hat{\alpha}_v, \hat{\beta}_v$, and $\hat{\gamma}_{iv}$ are calculated using their respective voxel-wise ordinary least squares estimates. $\hat{\sigma}_v^2$ is calculated as $\hat{\sigma}_v^2 = \frac{1}{N}\sum_{ij}(Y_{ijv} - (\hat{\alpha}_v + x_{ij}^T\hat{\beta}_v + \hat{\gamma}_{iv}))^2$, with $N = \sum_i n_i$.

Set $Z_{ij} = (Z_{ij1}, ..., Z_{ijV})$. We will assume $Z_{ij} \sim MVN(\gamma_i, \Sigma_i)$ and introduce a multivariate normal prior such that $\gamma_i \sim MVN(\gamma_{0i}, \tau_{0i}^2 I)$. We use the method of moments estimators to estimate $\gamma_i, \Sigma_i, \gamma_{0i}$, and τ_{0i}^2 such that

$$\hat{\gamma}_i = \frac{1}{n_i}\sum_j Z_{ij}, \quad \hat{\Sigma}_i = \frac{1}{n_i}\sum_j (Z_{ij} - \hat{\gamma}_i)(Z_{ij} - \hat{\gamma}_i)^T, \quad \hat{\gamma}_{0i} = \frac{1}{V}\mathbf{1}\mathbf{1}^T\hat{\gamma}_i, \quad \hat{\tau}_{0i}^2 = \frac{1}{V-1}(\hat{\gamma}_i - \hat{\gamma}_{0i})^T(\hat{\gamma}_i - \hat{\gamma}_{0i})$$

We can derive the conditional (posterior) distribution of $\gamma_i : \pi(\gamma_i|Z_i, \Sigma_i)$

$$\begin{aligned}
\pi(\gamma_i|Z_i,\Sigma_i) &\propto L(Z_i|\gamma_i,\Sigma_i)\pi(\gamma_i) \\
&= \exp\left\{-\tfrac{1}{2}\textstyle\sum_j (Z_{ij}-\gamma_i)^T\Sigma_i^{-1}(Z_{ij}-\gamma_i)\right\}\exp\left\{-\tfrac{1}{2\tau_{0i}^2}(\gamma_i-\gamma_{0i})^T(\gamma_i-\gamma_{0i})\right\} \\
&= \exp\left\{-\tfrac{1}{2}\left[\textstyle\sum_j (Z_{ij}-\gamma_i)^T\Sigma_i^{-1}(Z_{ij}-\gamma_i) + \tfrac{1}{\tau_{0i}^2}(\gamma_i-\gamma_{0i})^T(\gamma_i-\gamma_{0i})\right]\right\} \\
&= \exp\left\{\tfrac{-1}{2}\left[\textstyle\sum_j (Z_{ij}^T-\gamma_i^T)\Sigma_i^{-1}(Z_{ij}-\gamma_i) + \tfrac{1}{\tau_{0i}^2}(\gamma_i-\gamma_{0i})^T(\gamma_i-\gamma_{0i})\right]\right\} \\
&= \exp\left\{\tfrac{-1}{2}\left[\textstyle\sum_j (Z_{ij}^T\Sigma_i^{-1}-\gamma_i^T\Sigma_i^{-1})(Z_{ij}-\gamma_i) + \tfrac{1}{\tau_{0i}^2}(\gamma_i-\gamma_{0i})^T(\gamma_i-\gamma_{0i})\right]\right\} \\
&= \exp\left\{-\tfrac{1}{2}\left[\textstyle\sum_j (Z_{ij}^T\Sigma_i^{-1}Z_{ij}-2Z_{ij}^T\Sigma_i^{-1}\gamma_i+\gamma_i^T\Sigma_i^{-1}\gamma_i) + \tfrac{1}{\tau_{0i}^2}(\gamma_i-\gamma_{0i})^T(\gamma_i-\gamma_{0i})\right]\right\} \\
&= \exp\left\{\tfrac{-1}{2}\left[\textstyle\sum_j (Z_{ij}^T\Sigma_i^{-1}Z_{ij}-2Z_{ij}^T\Sigma_i^{-1}\gamma_i+\gamma_i^T\Sigma_i^{-1}\gamma_i) + \tfrac{1}{\tau_{0i}^2}(\gamma_i^T\gamma_i-2\gamma_{0i}^T\gamma_i+\gamma_{0i}^T\gamma_{0i})\right]\right\} \\
&\propto \exp\left\{(\tfrac{-1}{2}\left[\textstyle\sum_j (-2Z_{ij}^T\Sigma_i^{-1}\gamma_i+\gamma_i^T\Sigma_i^{-1}\gamma_i) + \tfrac{1}{\tau_{0i}^2}(\gamma_i^T\gamma_i-2\gamma_{0i}^T\gamma_i)\right]\right\} \\
&= \exp\left\{\tfrac{-1}{2}\left[\textstyle\sum_j (-2Z_{ij}^T\Sigma_i^{-1}\gamma_i+n_i\gamma_i^T\Sigma_i^{-1}\gamma_i) + \tfrac{1}{\tau_{0i}^2}(\gamma_i^T\gamma_i-2\gamma_{0i}^T\gamma_i)\right]\right\} \\
&= \exp\left\{\tfrac{-1}{2}\left[n_i\gamma_i^T\Sigma_i^{-1}\gamma_i + \tfrac{1}{\tau_{0i}^2}\gamma_i^T\gamma_i - 2\left(\textstyle\sum_j Z_{ij}^T\Sigma_i^{-1}\gamma_i + \tfrac{1}{\tau_{0i}^2}\gamma_{0i}^T\gamma_i\right)\right]\right\} \\
&= \exp\left\{\tfrac{-1}{2\tau_{0i}^2}\left[n_i\tau_{0i}^2\gamma_i^T\Sigma_i^{-1}\gamma_i + \gamma_i^T\gamma_i - 2\left(\tau_{0i}^2\textstyle\sum_j Z_{ij}^T\Sigma_i^{-1}\gamma_i + \gamma_{0i}^T\gamma_i\right)\right]\right\} \\
&= \exp\left\{\tfrac{-1}{2\tau_{0i}^2}\left[\gamma_i^T\left(n_i\tau_{0i}^2\Sigma_i^{-1}\right)\gamma_i + \gamma_i^T\gamma_i - 2\left(\tau_{0i}^2\textstyle\sum_j Z_{ij}^T\Sigma_i^{-1}\gamma_i + \gamma_{0i}^T\gamma_i\right)\right]\right\} \\
&= \exp\left\{\tfrac{-1}{2\tau_{0i}^2}\left[\gamma_i^T\left(n_i\tau_{0i}^2\Sigma_i^{-1}+I\right)\gamma_i - 2\left(\tau_{0i}^2\textstyle\sum_j Z_{ij}^T\Sigma_i^{-1} + \gamma_{0i}^T\right)\gamma_i\right]\right\} \\
&= \exp\left\{\tfrac{-1}{2\tau_{0i}^2}\left[\gamma_i^T\left(n_i\tau_{0i}^2\Sigma_i^{-1}+I\right)\gamma_i - 2\left(\tau_{0i}^2\Sigma_i^{-1}\textstyle\sum_j Z_{ij} + \gamma\right)^T\gamma_i\right]\right\}
\end{aligned}$$

bioRxiv preprint doi: https://doi.org/10.64898/2026.06.03.729396; this version posted June 8, 2026. The copyright holder for this preprint (which was not certified by peer review) is the author/funder, who has granted bioRxiv a license to display the preprint in perpetuity. It is made available under a CC-BY 4.0 International license.

By completing the square, we can conclude that

$$\pi(\gamma_i|Z_i,\Sigma_i) \propto \exp\left\{\frac{-1}{2}(\gamma_i-\tilde{\gamma_i})\,\Omega^{-1}\,(\gamma_i-\tilde{\gamma_i})\right\} \text{ where } \tilde{\gamma_i} = \left(n_i\tau_{0i}^2\Sigma_i^{-1}+I\right)^{-1}\left(\gamma_{0i}+\tau_{0i}^2\Sigma_i^{-1}\sum_j Z_{ij}\right)$$

with matrix Ω calculated accordingly. This shows the posterior distribution is a multivariate normal distribution. We can then estimate γ_i based on the posterior mean, resulting in the following estimate:

$$\gamma_i^* = \left(n_i\hat{\tau}_{0i}^2\hat{\Sigma}_i^{-1}+I\right)^{-1}\left(\hat{\gamma}_{0i}+\hat{\tau}_{0i}^2\hat{\Sigma}_i^{-1}\sum_j Z_{ij}\right)$$

To model the batch-specific covariance effects, we first estimate the overall pooled covariance based on the sample covariance estimate: $\hat{\Sigma} = \frac{1}{N-1}\sum_{i,j}(Z_{ij}-\hat{\gamma})(Z_{ij}-\hat{\gamma})^T$, where $N = \sum_i n_i$ and $\hat{\gamma} = \frac{1}{N}\sum_{ij} Z_{ij}$. We then assume that $\Sigma_i = A_i\Sigma A_i^T$, where A_i is a matrix dictating the batch-specific rotation. Leveraging the eigendecomposition of real symmetric matrices, we can rewrite the covariance matrices as $\Sigma_i = L_iL_i^T$ and $\Sigma = PP^T$, in which

$$\begin{aligned} L_i &= UD^{1/2}, \text{where } U \text{ is a matrix of eigenvectors and } D^{1/2} = diag(\sqrt{\lambda_1},...,\sqrt{\lambda_V}) \text{ of } \hat{\Sigma}_i \\ P &= EK^{1/2}, \text{where } E \text{ is a matrix of eigenvectors and } K^{1/2} = diag(\sqrt{\lambda_1},...,\sqrt{\lambda_V}) \text{ of } \hat{\Sigma} \end{aligned}$$

with $\lambda_1,...,\lambda_V$ as the eigenvalues of the respective covariance matrix. We can then write that $L_iL_i^T = A_iPP^TA_i^T$ and subsequently $A_i = L_iP^{-1}$. We finally adjust the data as follows to get the harmonized vector of imaging features M_{ij}

$$M_{ij} = \hat{\sigma}A_i^{-1}(Z_{ij}-\gamma_i^*)+\hat{\alpha}+X_{ij}\hat{\beta},$$

where $\hat{\alpha} = (\hat{\alpha}_1,...\hat{\alpha}_V)^T$, $\hat{\beta} = (\hat{\beta}_1,...\hat{\beta}_V)^T$, $\hat{\sigma} = diag(\hat{\sigma}_1,....,\hat{\sigma}_v)$, and X_{ij} is the matrix of covariate information.

bioRxiv preprint doi: https://doi.org/10.64898/2026.06.03.729396; this version posted June 8, 2026. The copyright holder for this preprint (which was not certified by peer review) is the author/funder, who has granted bioRxiv a license to display the preprint in perpetuity. It is made available under a CC-BY 4.0 International license.

5.2 Supplemental Simulation Results: Medium Difference

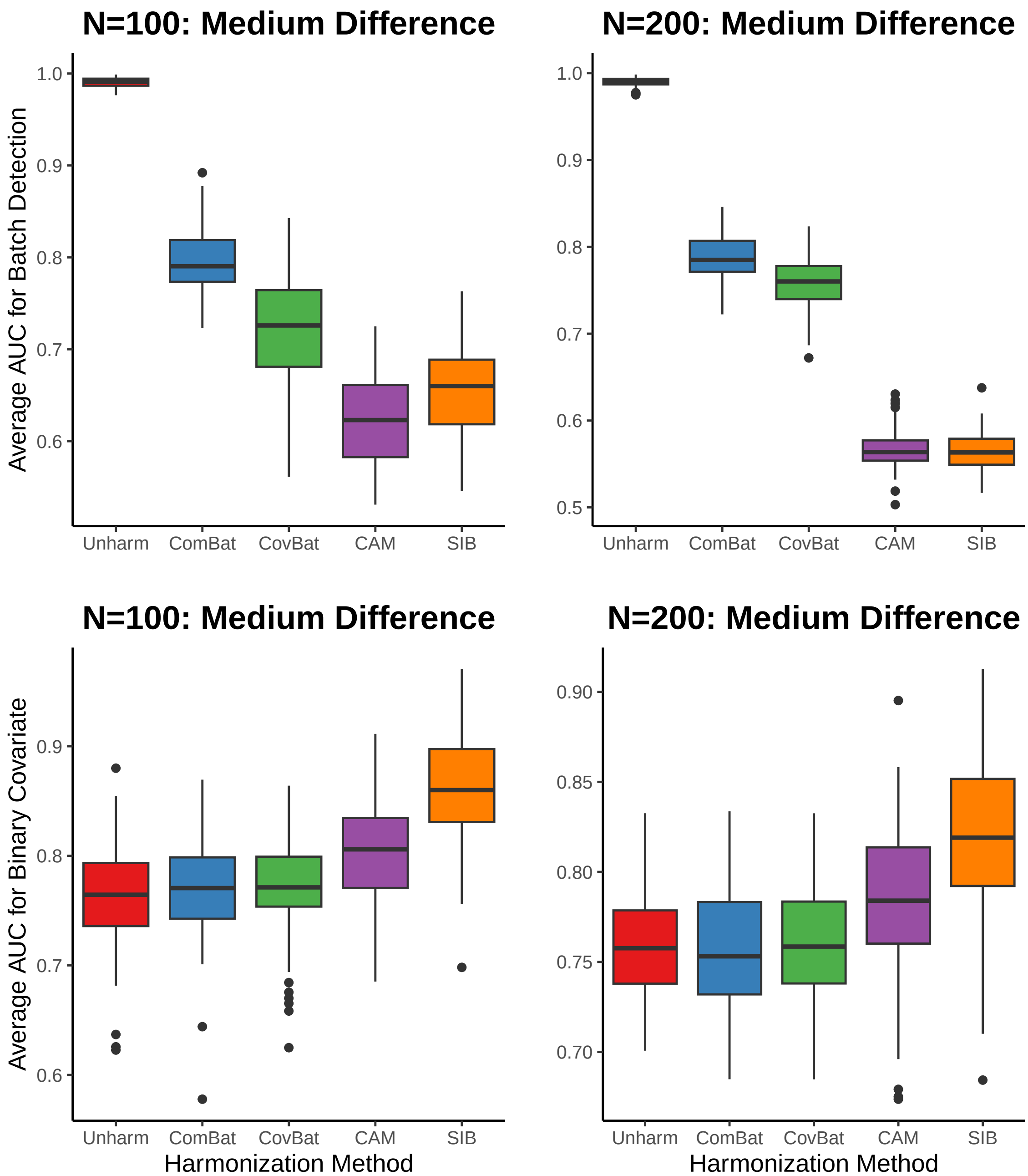

Figure 9: Side-by-side boxplots comparing AUC for detecting batch (top row) and binary covariate level (bottom row) between ComBat, CovBat, CAM ComBat, and SIB ComBat. Comparisons done between N=100 (left) and N=200 (right) for medium difference between covariances.

bioRxiv preprint doi: https://doi.org/10.64898/2026.06.03.729396; this version posted June 8, 2026. The copyright holder for this preprint (which was not certified by peer review) is the author/funder, who has granted bioRxiv a license to display the preprint in perpetuity. It is made available under a CC-BY 4.0 International license.

5.3 Supplemental Figure: The Effect of Different Harmonization Methods on Age Association

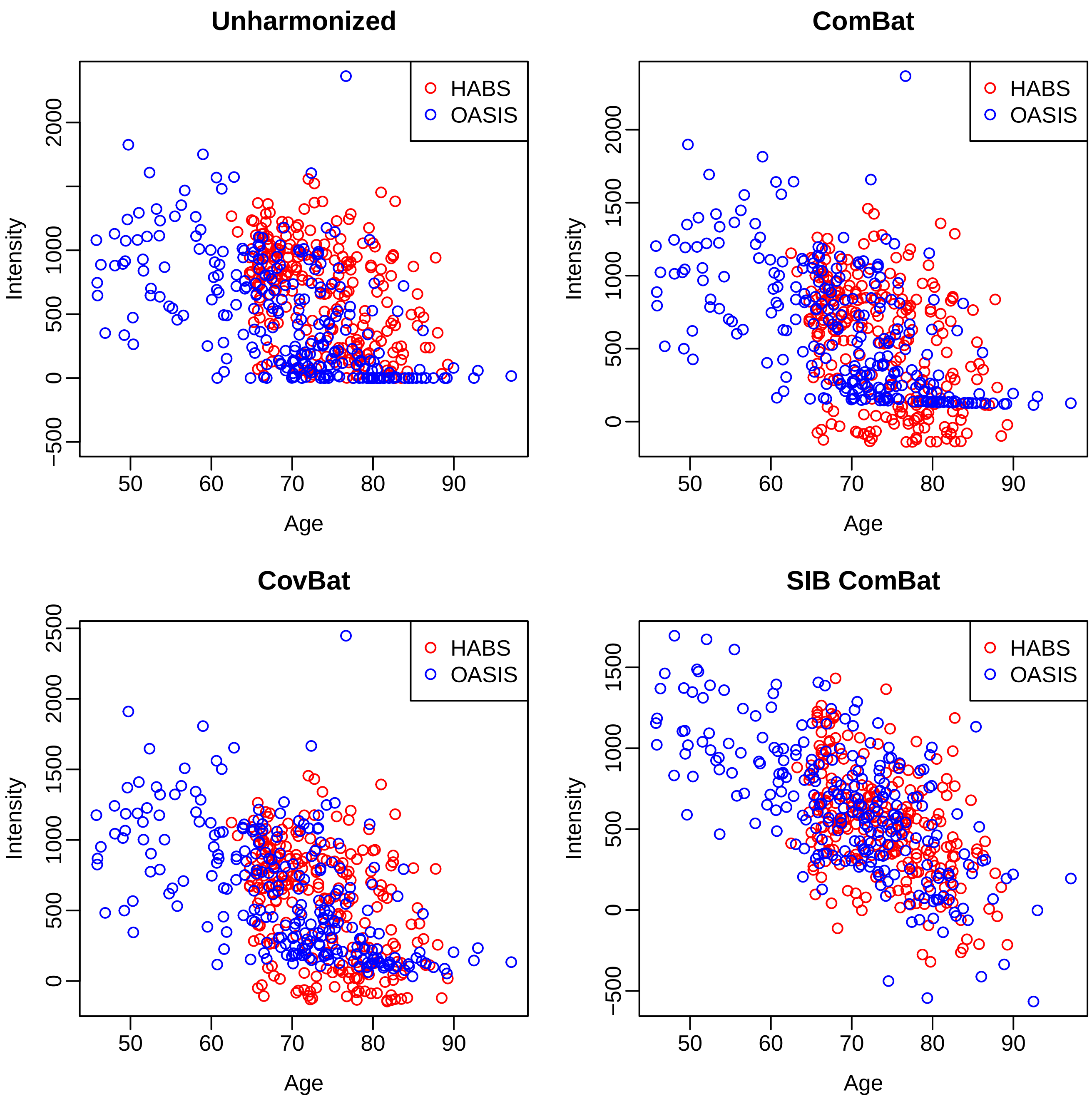

Figure 10: Scatter plots visualizing the association between the intensity of a given voxel-level feature and age across different harmonized datasets. Associations are compared between individuals from HABS against those from OASIS.

bioRxiv preprint doi: https://doi.org/10.64898/2026.06.03.729396; this version posted June 8, 2026. The copyright holder for this preprint (which was not certified by peer review) is the author/funder, who has granted bioRxiv a license to display the preprint in perpetuity. It is made available under a CC-BY 4.0 International license.

5.4 Supplemental VBM Analysis

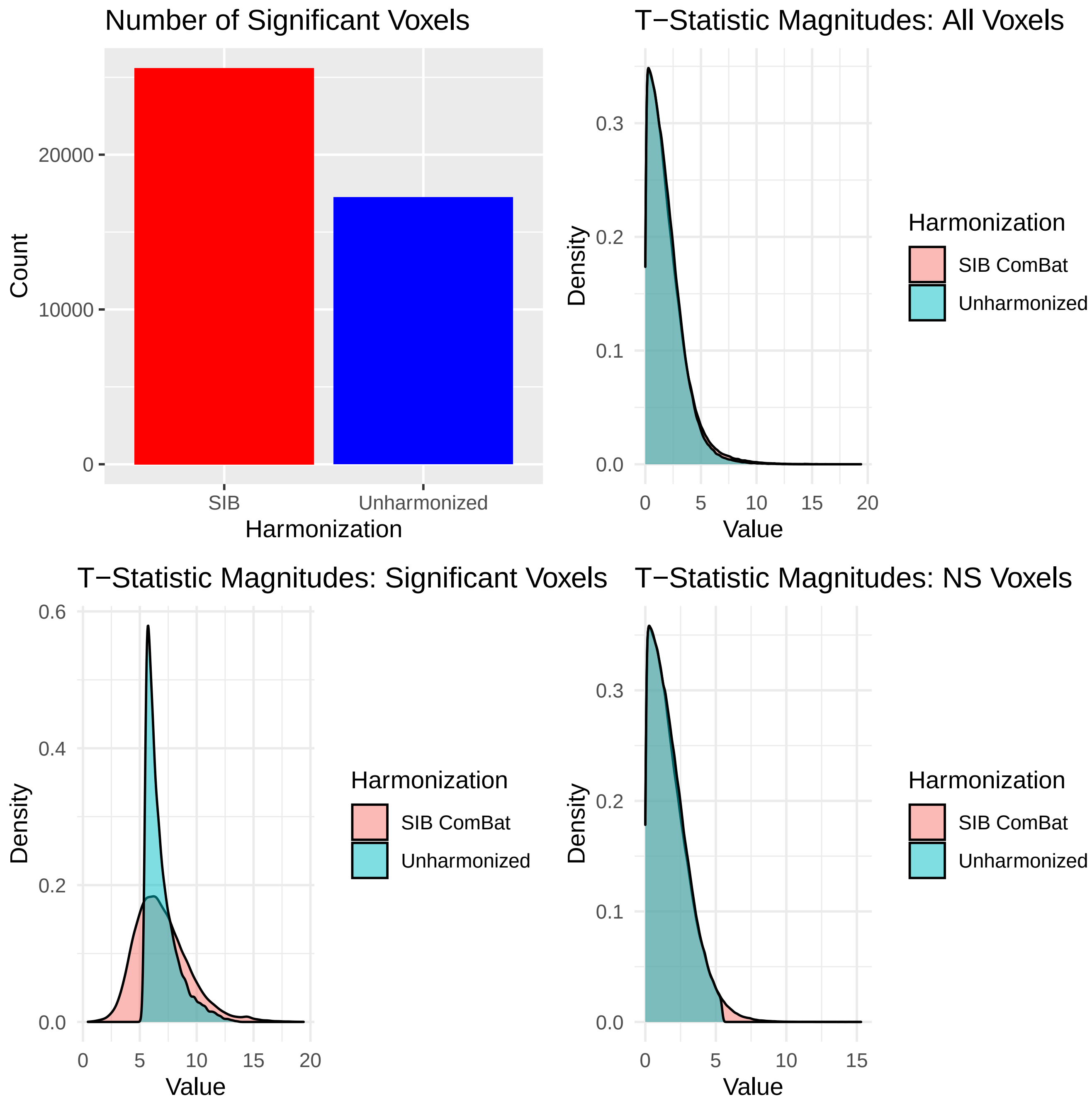

Figure 11: Plots showing the number of significant voxels for age (upper left) and the distributions of age t-statistic magnitudes across all voxels (upper right) / originally significant voxels (lower left) / originally not significant (NS) voxels (lower right) between SIB ComBat-harmonized and unharmonized data.

bioRxiv preprint doi: https://doi.org/10.64898/2026.06.03.729396; this version posted June 8, 2026. The copyright holder for this preprint (which was not certified by peer review) is the author/funder, who has granted bioRxiv a license to display the preprint in perpetuity. It is made available under a CC-BY 4.0 International license.

5.5 Supplemental Figure: Comparison of Principal Component Loadings Between Sites

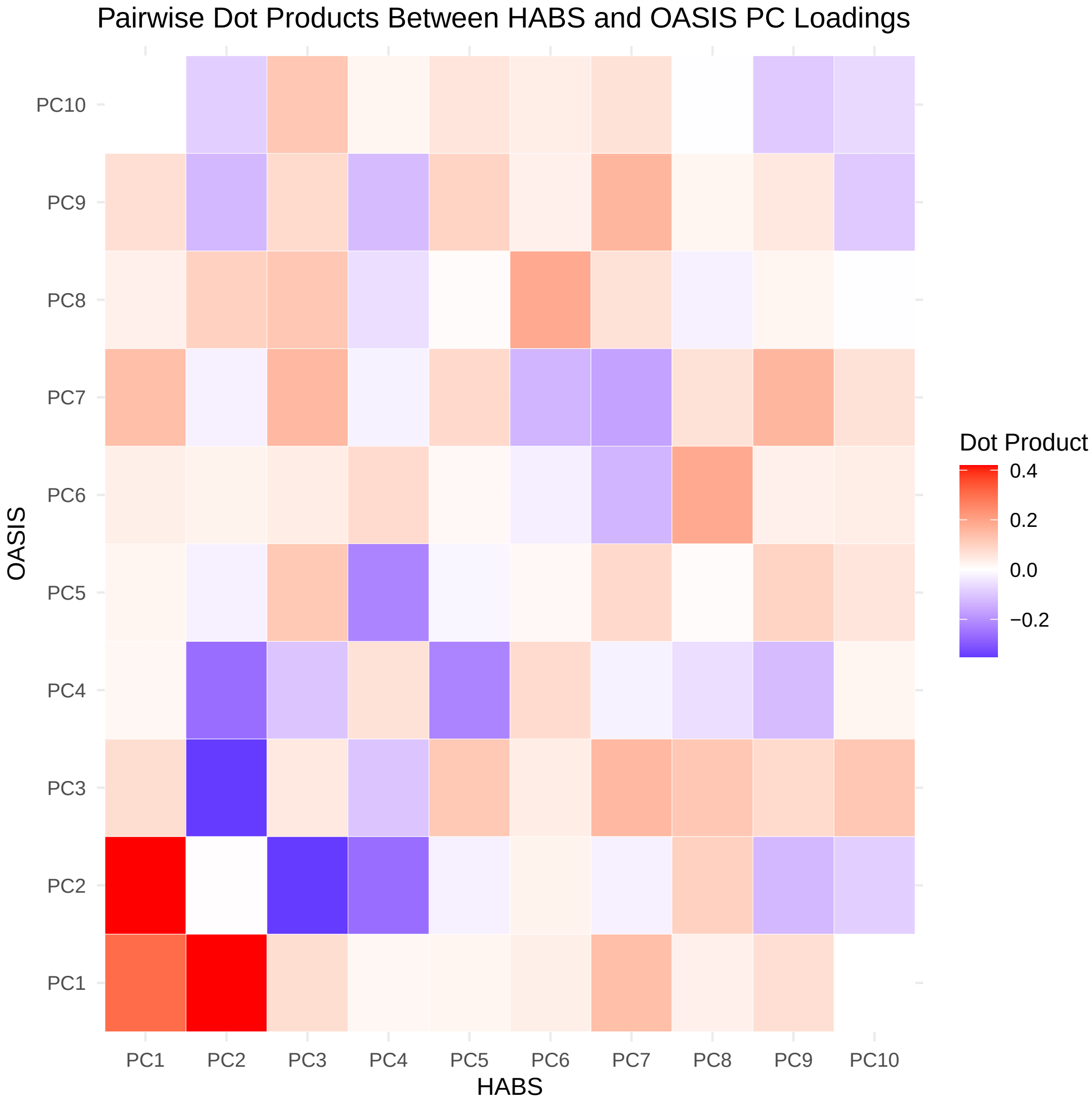

Figure 12: Heatmap that illustrates the pairwise similarity of the first ten principal component eigenvectors between two study sites, HABS and OASIS, by calculating their respective dot product. In this figure, most of the dot products between eigenvectors were close to 0, demonstrating strong dissimilarity.
